# A young gas giant and hidden substructures in a protoplanetary disk

**DOI:** 10.1038/s41550-025-02576-w

**Published:** 2025-07-14

**Authors:** Álvaro Ribas, Miguel Vioque, Francesco Zagaria, Cristiano Longarini, Enrique Macías, Cathie J. Clarke, Sebastián Pérez, John Carpenter, Nicolás Cuello, Itziar de Gregorio-Monsalvo

**Affiliations:** 1 Institute of Astronomy, University of Cambridge, Cambridge, UK; 2 European Southern Observatory, Garching bei München, Germany; 3 Max Planck Institute for Astronomy, Heidelberg, Germany; 4 Millennium Nucleus on Young Exoplanets and their Moons, Santiago, Chile; 5 Departamento de Física, Universidad de Santiago de Chile, Santiago, Chile; 6 Center for Interdisciplinary Research in Astrophysics and Space Science, Universidad de Santiago de Chile, Santiago, Chile; 7 Joint ALMA Observatory, Santiago, Chile; 8 Univ. Grenoble Alpes, CNRS, IPAG, Grenoble, France; 9 European Southern Observatory, Santiago, Chile

**Keywords:** Astrophysical disks, Exoplanets

## Abstract

The detection of planets in protoplanetary disks has proven to be extremely challenging. By contrast, rings and gaps, usually attributed to planet–disk interactions, have been found in virtually every large protoplanetary (Class II) disk observed at 0.9–1.3 mm with sufficient spatial resolution (5 au). The nearby disk around MP Mus (PDS 66) stands as an exception to this rule, and its advanced age (7–10 Myr) is particularly difficult to reconcile with its apparent lack of substructures. Despite the disk’s smooth appearance, Gaia data of MP Mus show a significant proper motion anomaly, signalling the presence of a companion. Here we present ALMA 3-mm observations of the system with high spatial resolution comparable to previous 1.3-mm data. The new observations pierce deeper into the disk midplane and reveal an inner cavity (<3 au) and a ring at 10 au. The disk structure inferred from ALMA observations narrows down the properties of the companion to a gas giant orbiting at 1–3 au, and hydrodynamic simulations further confirm that such a planet can produce the observed cavity. These independent pieces of evidence constitute an indirect but compelling detection of an exoplanet within a protoplanetary disk using Gaia astrometry. The detection of dust substructures in MP Mus, thanks to the lower optical depths at longer wavelengths, suggests that rings and gaps are even more abundant than previously thought.

## Abstract (web summary)

ALMA and Gaia data combine to reveal a young gas giant in the protoplanetary disk around MP Mus. Previously unseen substructures, visible only at longer wavelengths, suggest that more planets may hide in disks than previously thought.

## Main

Despite extensive efforts to find protoplanets in protoplanetary disks, they have been robustly detected in only two systems so far (PDS 70 and IRAS 04125+2902)^1–3^. Accessing the population of young planets would be truly valuable for planet formation studies as this would provide direct benchmarks to our models, but common detection methods such as transits, radial velocity or direct imaging are extremely challenging or simply unfeasible for embedded companions owing to the high dust extinction in the disk and the increased stellar activity of young stars (for example, refs. ^4,5^). Instead, the presence of planets in disks is typically inferred by planet–disk interaction footprints such as disk substructures and velocity perturbations. In fact, arguably the most important discovery of the past decade for the field of planet formation is the ubiquity of substructures in protoplanetary disks. Rings, gaps, spiral arms or azimuthal asymmetries are found in virtually every large disk observed with sufficient angular resolution^6–8^.

Disk substructures offer a natural solution to the problem of dust radial drift: the interaction of millimetre-to-centimetre-sized grains with the gas in the disk causes large grains to drift towards the central star. This process is much faster than typical timescales for planet formation and can quickly deplete the population of large grains in disks, presenting a theoretical barrier to build rocky planets and the cores of gas giants^9,10^. Local gas pressure maxima (induced by planets or other processes) have been proposed as a mechanism to trap large grains and allow them to grow to larger sizes^11–13^, but this solution remained hypothetical until the high resolution and sensitivity of observatories such as the Atacama Large Millimeter/Submillimeter Array (ALMA) and the Spectro-Polarimetric High-contrast Exoplanet REsearch instrument (SPHERE) uncovered the plethora of disk substructures (for example, refs. ^6,14,15^).

As of today, MP Mus is the only large Class II protoplanetary disk that appears structureless when observed at millimetre wavelengths with high spatial resolution^16^ (<5 au). This K1V star is located 97.9 ± 0.1 pc away from Earth^17^ and has an estimated age of 7–10 Myr (ref. ^16^). Its dust disk extends up to *R*_dust,90%_ = 45 au at 1.3 mm (that is, the radius encompassing 90% of the 1.3-mm continuum emission), while the gaseous disk traced by the ^12^CO(J=2-1) reaches *R*_CO,90%_ = 110 au. This ratio of gas to dust radii is similar to that of structured disks, in which dust traps are probably acting^18^. However, the disk appears structureless at 1.3 mm down to 4 au scales except for a tentative narrow dust ring in the outer regions^16^, recently confirmed by ref. ^19^ in high-resolution ALMA 0.89 mm observations. By contrast, scattered light observations of this system show an apparent gap between 40 au and 80 au (refs. ^20,21^). Nevertheless, the fact that the millimetre disk extends up to ~55 au, the clearly Keplerian rotation of the disk as traced by ALMA and its flat appearance strongly suggest that the drop in scattered light intensity between 40 au and 80 au is due to a shadowing effect and not an actual gap in the disk^16^. The smooth 1.3-mm radial profile is yet more puzzling when we compare the characteristic lifetime of disks^22–24^ (~3 Myr) with the age of MP Mus. To explain the survival of large dust grains in such an old and seemingly structureless disk, ref. ^16^ proposed a number of possible solutions: (1) substructures may be present in the disk but the 1.3-mm continuum is optically thick, effectively hiding them; (2) substructures smaller than 4 au exist and remain unresolved with current observations; or (3) the disk is really featureless and a different (unknown) mechanism is retaining millimetre-emitting grains in it. None of these scenarios could be tested with the existing observations at the time, and MP Mus remained an outlier among disks.

## The Gaia proper motion anomaly of MP Mus

The 1.3-mm continuum emission of MP Mus shows no obvious signs of planets in the system but, by contrast, its astrometry deviates from a single star solution. By measuring the proper motion vector of the photocentre at different times, we can identify changes (proper motion anomalies, PMas) that signal the presence of companions^25–27^. PMas have already been used to identify exoplanets around main-sequence stars (later confirmed with high-resolution imaging, for example, refs. ^28–31^), but this method has not been applied to sources with protoplanetary disks yet. Compared with main-sequence stars, protostars exhibit additional phenomena (for example, variability, strong disk asymmetries, differential extinction and stellar obscuration by accretion columns) that could potentially induce a PMa. However, the analysis of well-known protoplanetary disks shows that only edge-on, highly asymmetric or massive disks can induce PMas (Vioque et al., in prep), and MP Mus has a very low accretion rate^32^ (~10^−10^ *M*_⊙_ yr^−1^). Therefore, none of these potential issues applies to the MP Mus system^16^.

We determined the PMa of MP Mus by comparing the proper motion vectors of Gaia DR2 and Gaia DR3 (for details on the technique, see refs. ^33,34^). The resulting PMa has a significance of 4.5*σ* (where *σ* is the uncertainty of the PMa) and implies a change in the proper motion of ∣Δ*μ*∣ = 0.21 ± 0.05 mas yr^−1^. We used the scanning law of Gaia DR2 (ref. ^35^) and Gaia DR3 (ref. ^36^) to retrieve the epochs and scan angles in which Gaia observed this source. We then used these epochs and angles, together with realistic Gaia uncertainties, to simulate the Gaia DR2 and DR3 observations of MP Mus assuming a two-body system with different mass ratios and periods [using the astromet package developed by ref. ^33^). We assumed no eccentricity and a relation between the mass (*q* = *M*_comp_/*M*_primary_) and light (*l* = *L*_comp_/*L*_primary_) ratio of the components of *l* = *q*^3.5^, a heuristic commonly used for main-sequence stars. We ran 500,000 simulations randomly sampling the log-uniform space of mass ratios (from 0.0001 to 1) and periods (from 0.016 to 1,585 years). The mass of the primary source, the viewing angles and the orbital phase were sampled at random every time following the measured values and uncertainties of stellar mass (1.30 ± 0.08 *M*_⊙_), inclination (32 ± 1°) and position angle (10 ± 1°, disk geometries from ref. ^16^). We then selected only those simulations that resulted in astrometric properties compatible with what is observed in MP Mus (0.26 > ∣Δ*μ*∣ > 0.16 mas yr^−1^, ∣Δ*μ*∣/*σ*_∣Δ*μ*∣_ > 4). In addition, MP Mus has a renormalized unit weight error (RUWE) very close to 1 in both Gaia DR2 and DR3 (of 0.98 and 0.96, respectively). We hence imposed a conservative threshold to the simulations of UWE_DR3_ <1.1 (see section 4.1 of ref. ^33^).

**Fig. 1:**
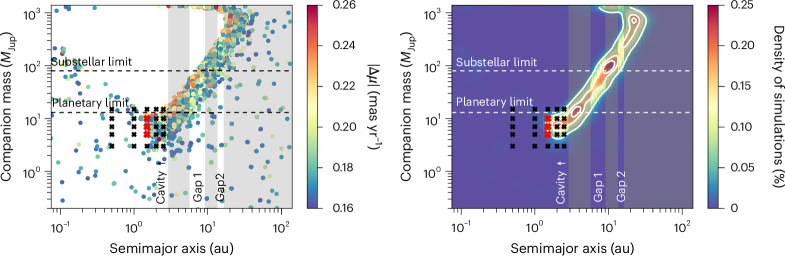
Orbital separation and mass of a companion that could produce the Gaia proper motion anomaly of MP Mus. Left: the individual Gaia simulations that produce an astrometric signal compatible with the observations (0.26 > ∣Δ*μ*∣ > 0.16 mas yr^−1^, ∣Δ*μ*∣/*σ*_∣*Δμ*∣_ > 4 and UWE_DR3_ <1.1). Right: the same simulations but smoothed over a two-dimensional histogram (contours correspond to 0.05%, 0.15% and 0.2%). When considering the disk structure traced in the millimetre (shaded area), the most likely companion is a gas giant at 1–2 au. Crosses indicate the parameters used for the hydrodynamical simulations in the ‘Confronting observations with simulations’ section. The simulations that best reproduce both Gaia and ALMA observations are shown in red (Discussion).

Figure 1 shows the orbital separations and masses for a companion to induce the observed PMa and RUWE. We note that we model only a two-body interaction, and the interpretation of the PMa becomes more complex for multibody systems. In this work, we assume that, although there may be other planets in MP Mus, its PMa is dominated by a single companion. From the Gaia data, we conclude that the PMa of MP Mus is most likely to be due to a companion at orbital separations <30 au, with the required mass increasing with orbital distance (Fig. 1). The non-detection of astrometric noise (that is, RUWE ~1) also rules out massive bodies with short periods (that is, *a* < 1 au). The strong statistical significance of the Gaia PMa detection makes for a good companion candidate in MP Mus, but a second, independent signature is needed to further constrain its location and mass.

## Hidden substructures at 3 mm

To better understand MP Mus, we obtained new ALMA observations at 3 mm and with a spatial resolution (4 au) similar to that of the 1.3-mm data presented by ref. ^16^ (see the Methods for details about the observations and data processing). Longer wavelengths offer two main advantages to search for substructures in disks. First, the dust opacity (and therefore the optical depth) decreases with wavelength, and the observations probe emission closer to the disk midplane where planets are expected to form. This is most important in the disk inner regions where the emission is likely to be optically thick, as indicated by the low (*α* < 2) local millimetre spectral index of MP Mus between 1.3 mm and 2.2 mm (ref. ^16^). A second benefit of observations at longer wavelengths is that they trace larger grains, which experience a stronger gas drag and are more strongly trapped in pressure maxima. Therefore, if present, substructures may show an enhanced contrast at longer wavelengths (for example, ref. ^37^).

**Fig. 2:**
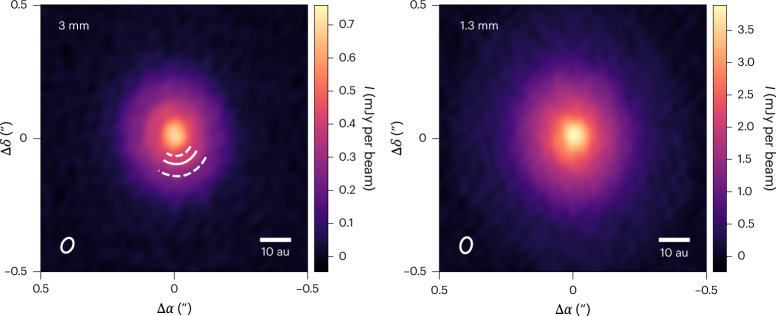
ALMA continuum observations of MP Mus at 3 mm and 1.3 mm. The solid and dashed white arcs in the 3-mm image mark the ring and two gaps. The beam of each observation is indicated as a white ellipse in the bottom left corners.

**Fig. 3:**
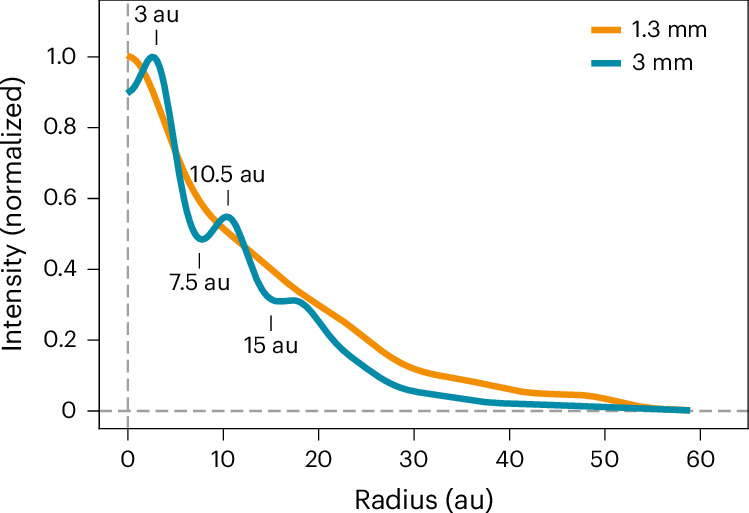
Intensity radial profiles of MP Mus derived using FRANK. The 3-mm profile shows structures undetected at shorter wavelengths, including an inner cavity, a ring and two gaps. These features are indicated in the figure.

The ALMA 3-mm observations are shown in Fig. 2 alongside previous 1.3-mm data. The disk appears smooth at 1.3 mm (ref. ^16^), but the new 3-mm data reveal a ring at 10.5 au and two gaps at 7.5 and 15 au that are undetected at shorter wavelengths. The 3-mm image also has flatter central emission, with hints of two peaks suggesting the presence of a barely resolved cavity (this is seen more clearly in Supplementary Fig. 1). To better characterize the structure of the system, we calculated and compared the intensity radial profiles of the disk at both wavelengths. We do this in visibility space with the code FRANK^38^, which achieves higher angular resolution and recovers smaller structures than working with synthesized images. The profiles are shown in Fig. 3 and more clearly reveal the aforementioned structures in the 3-mm image: an inner cavity with a radius of ~3 au, as well as a ring and two gaps. The FRANK residuals are provided in Supplementary Fig. 2. Although not as clear, radial profiles calculated directly from synthesized images also show these substructures (Methods and Supplementary Fig. 3).

The 3-mm data prove that MP Mus is indeed structured, which could explain both the disk longevity and the similar ratio of gas/dust radii to other structured disks. A naive interpretation of the disk architecture, in which the inner cavity and two gaps are each being carved by a different planet, would imply that MP Mus hosts a multiplanetary system. Nevertheless, we caution that mechanisms other than planets can open cavities and gaps^39–41^ and that such interpretation may be too simplistic. Moreover, simulations have shown that a single planet may carve multiple gaps in low-viscosity disks^42,43^, although this is no longer the case for models including radiative effects^44^.

We can, however, attempt to link the new-found structures to the Gaia PMa to search for possible explanations for both phenomena. First, previous observing campaigns to detect planets and substellar objects in MP Mus have ruled out sources more massive than 2–3 *M*_Jup_ at separations >40–50 au (for example, refs. ^20,45^). If instead we assume that a putative companion in either of the gaps (that is, at 7.5 au or at 15 au) is responsible for the PMa, it would require masses above 10 *M*_Jup_ to account for the observed signal (Fig. 1). Such a massive object would open much wider gaps and strongly alter the disk structure, in stark contrast to its narrow gaps and overall smooth appearance (by comparison, the vast majority of planetary masses determined from gap properties are well below 3 *M*_Jup_)^8,46^. For these reasons, even if planets may be carving those gaps, they are unlikely to be the cause of the PMa. Meanwhile, the inner ~3-au cavity appears as an optimal location for possible companions. We can further restrict the location of the companion if we assume that it is responsible for carving the cavity: even in the most favourable scenario (that is, an equal mass binary), a non-eccentric binary system can open a cavity only as large as two to three times the orbital separation^47,48^. Put together, the Gaia and ALMA data strongly suggest that MP Mus harbours a giant protoplanet in the inner 1–3 au of the system.

## Confronting observations with simulations

**Fig. 4:**
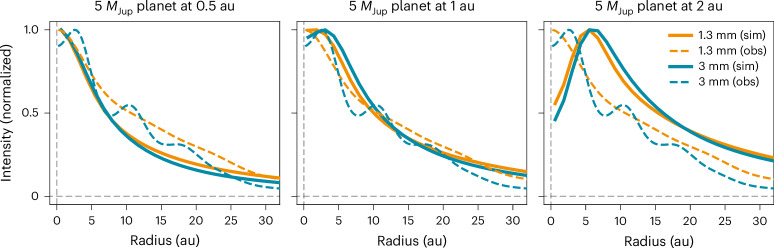
Intensity radial profiles for three of the SPH simulations. The simulation (sim) profiles are shown as solid lines at 1.3 mm (orange) and 3 mm (blue), after convolution with the beams of the ALMA observations. The observed (obs) profiles are also shown as dashed lines. The panels show a 5 *M*_Jup_ planet at 0.5 au (left), 1 au (middle) and 2 au (right). In the first case, the cavity opened by the planet is too small to be resolved in the observations. Planets with separations ≥2 au produce cavities that are too large to be compatible with the data.

To explore if a protoplanet within the mass range and orbital separations suggested by the Gaia PMa could explain the inner cavity seen at 3 mm, we ran hydrodynamical simulations of a star + planet system using the smoothed particle hydrodynamics (SPH) code PHANTOM^49^. We ran a grid of 25 models with different orbital radii and masses that would fit inside the 3-au cavity, namely at 0.5, 1, 1.5, 2 and 2.5 au (corresponding to orbital periods of 0.3, 0.9, 1.6, 2.5 and 3.5 years, respectively), and with companion masses of 3, 5, 7, 10 and 15 *M*_Jup_. Details on the modelling set-up are provided in the Methods. We then performed radiative transfer with the MCFOST code^50,51^ to produce continuum images at 1.3 and 3 mm and convolved the results with the corresponding ALMA beams. Figure 4 shows the radial profiles of the simulations at 1.3 and 3 mm for three representative cases as calculated directly from the images with the code GoFish^52^. The results for the full grid are provided in Supplementary Information section 1.2 and Supplementary Fig. 4.

The SPH simulations provide additional information about the properties of the companion. First, none of the models with an orbital separation of 0.5 au carve an inner cavity large enough to be discernible with the current spatial resolution, regardless of their mass. On the other extreme, planets at ≥2 au produce cavities that are too large to be compatible with the observations. The mass of the companion does not have a strong effect on the cavity size, but more massive objects result in deeper cavities. We also note that some simulations create a cavity of the right size at 3 mm while no cavity is visible at 1.3 mm, just as observed with ALMA (although this is more sensitive to the adopted disk and dust properties). Overall, the simulations show that the observed cavity can be successfully explained by a gas giant orbiting at 1–2 au, lending further indirect but independent support to the presence of such a planet in MP Mus.

## Discussion

The combination of two independent but consistent pieces of evidence (the Gaia PMa and the finding of a small inner cavity) create a very compelling case for the existence of MP Mus b, a gas giant planet orbiting between approximately 1 au and 3 au. The case is strengthened even further when we consider the SPH simulations of the system, which show that planets between 1 au and 2 au carve a cavity with the correct size and in some cases even reproduce the peculiar feature of the inner cavity being visible at 3 mm but not at 1.3 mm. The presence of a companion in the inner regions of MP Mus would also account for its low accretion rate compared with other disks with similar masses^32^ (~10^−10^ *M*_⊙_ yr^−1^). It is unlikely that such a planet would form at its current location: planet formation is thought to be more efficient at radii near and outside the snowline (the location at which water ice sublimates) owing to the change in dust properties and enhanced dust-to-gas ratios (for example, ref. ^53^), and the gas giants found with 1–2 au orbits are believed to have undergone planetary migration (for example, ref. ^54^). Interestingly, at 1–2 au, it is possible that MP Mus b resides in the habitable zone of its 1.3 *M*_⊙_ star. Detecting such a planet with radial velocity would be a formidable challenge, as the expected signal of a ~5 *M*_Jup_ between 1 au and 3 au (that is, a radial velocity semi-amplitude of 40–70 m s^−1^) is below the typical level of stellar activity in young stars (~100 m s^−1^, for example, ref. ^55^), and even detections of very massive protoplanets much closer to their host stars are very difficult to disentangle from other sources of radial velocity signal (see the case of CI Tau^5,56–59^). This highlights the unique potential of the PMa technique to uncover the population of young planets in close (a few astronomical units) orbits embedded in protoplanetary disks, a region of the parameter space that remains beyond the reach of other current detection methods. This becomes especially promising when combined with additional information about the possible presence of the protoplanet such as in the case of MP Mus, where the disk structure traced by ALMA allows one to pinpoint the location of the companion; the narrow width of the two gaps rules them out as possible sites for the body inducing the PMa, leaving the inner cavity as the only suitable location.

In addition to the inner cavity revealed by the new 3-mm data, the different morphology of the disk at 1.3 and 3 mm can be attributed solely to the difference in wavelength (given that both observations have the same spatial resolution). As mentioned in the ‘Hidden substructures at 3 mm’ section, this arises from a combination of the enhanced contrast of substructures (due to more efficient trapping of larger grains) and the lower optical depth with increasing wavelength. Previous studies have already shown that disk substructures often appear sharper and with a higher contrast at longer wavelengths (for example, refs. ^60–62^). MP Mus represents an extreme example of this as it is the first source in which new substructures are uncovered at longer wavelengths, but it is unlikely to be the only system in which this occurs. The new 3-mm observations also confirm that 1-mm emission from the inner regions of protoplanetary disk can remain optically thick and, more importantly, indicate that our current census of disk substructures (see ref. ^8^ and references therein) is potentially very incomplete. Protoplanetary disks may be more structured than currently believed, on the one hand providing even more dust trapping of large grains and on the other hand clearly necessitating high-resolution, high-sensitivity surveys at longer wavelengths to understand the frequency and properties of a potential population of hidden substructures.

## Methods

### ALMA data processing and imaging

The MP Mus ALMA Band 3 (3 mm) data correspond to the ALMA project 2022.1.01758.S (principal investigator (PI): Á. Ribas) and were taken with two different configurations. The compact configuration was observed once on 21 May 2022 and contains baselines from 27 m to 3.6 km. The extended configuration was observed three times on 27–28 July 2022 with baselines ranging from 230 m to 16.2 km. The correlator was set up to maximize the continuum sensitivity, containing four spectral windows centred at 90.521, 92.416, 102.521 and 104.479 GHz, each with a bandwidth of 1.875 GHz and 128 channels. All the observations were first calibrated using the pipeline script and CASA v.6.4.1.12, and further processing was performed with CASA v.6.5.6.22. In particular, we first performed phase-only self-calibration on the extended and compact configurations separately. We then placed the phase centre on the disk for each observation, set the coordinates of each phase centre to a common value and rescaled the flux of the three extended executions to match the flux of the compact one. Finally, one additional round of phase-only self-calibration was applied after combining the four execution blocks.

The Band 6 data (1.3 mm) are part of ALMA project 2017.1.01167.S (PI: S. Pérez) and were already presented in ref. ^16^. That work^16^ combined these observations with those of ALMA project 2017.1.01419.S (PI: C. Cáceres), which had lower spatial resolution but were useful to study gas emission in the system. We chose not to include those data in this work because here we focus on the continuum only. The data consist of a compact configuration observed on 15 January 2018 with baselines between 15 m and 2.4 km and an extended one taken on 16 November 2017 with baselines between 90 m and 8.2 km. The observations were calibrated with the pipeline and CASA v.5.1.1-5, and we then used CASA v.6.5.6.22 for the rest of the analysis. The correlator set-up in this case contained four spectral windows, three for continuum centred at 232.483, 244.983 and 246.983 GHz (bandwidth of 1.875 GHz and 128 channels each) and the fourth one centred at 230.525 GHz targeting the ^12^CO (2–1) line (bandwidth of 1.875 GHz and 960 channels). We flagged channels containing CO emission and then performed self-calibration with a similar process as for the Band 3 data: we first self-calibrated the compact and extended configurations individually, placed the phase centre at the centre of the disk in each case and changed its coordinates to a common value, and then rescaled the compact configuration to have the same flux as the extended one (in this case, the observatory calibrator appeared more stable around the time of the extended observations, although this has no impact on any of our results). We then ran one final round of phase-only self-calibration in which both the compact and extended configurations were combined.

We imaged the data using the tclean routine in CASA using different weighting to achieve the best compromise between spatial resolution and sensitivity. For the purpose of analysing the disk structures (Fig. 2), we used the mtmfs option with nterms = 1 and scales of 0, 1, 3 and 5 beams. We adopted a robust parameter of 0 for both wavelengths, resulting in a beam of 0.06″ × 0.04″ in both cases, and a position angle (PA) of −13° at 1.3 mm and −27° at 3 mm. The corresponding image root mean square (RMS) values are 37 μJy per beam and 7.7 μJy per beam at 1.3 mm and 3 mm, respectively. The peak signal-to-noise ratio (S/N) of these images is 105 at 1.3 mm and 90 at 3 mm. The disk flux, radius and mass measured at 3 mm are provided in Supplementary Information section 1.

### Radial profiles and additional sanity checks

The radial profiles in Fig. 3 were created using the FRANK code^38^. Under the assumption that the the disk is axisymmetric, this software calculates the radial profile of the disk directly from the observed visibilities using a Hankel transform. For both the 1.3- and 3-mm data, we first fit the geometry of the system with the non-parametric option and using the measured disk inclination (*i*)  and position angles as initial guesses^16^ (*i* = 32°, PA 10°). The values of *i* and *PA* determined by FRANK are compatible with the 1° uncertainty of these quantities. We then explored the effect of changing the *α* and *w*_smooth_ hyperparameters: the results were largely insensitive to the choice of these in the case of the 1.3-mm data, yielding a profile similar to the one presented in ref. ^16^ (unless extreme values of *α* and *w*_smooth_ were used, which induced artificial wiggles), and we adopted *α* = 1.2 and *w*_smooth_ = 5 × 10^−3^. In the case of 3 mm, we found that going below *α* = 1.3 or *w*_smooth_ = 10^−2^ produced clearly artificial ripples in the profile, and we thus used these values. We then imaged the residuals (subtracting the FRANK visibilities from the observed ones), which appear largely signal-free (Supplementary Fig. 2): the residuals are below three times the image noise (RMS) in the case of the 3-mm observations, and the 1.3 mm show only two localized blobs around the 5 RMS level.

We performed two sanity checks to ensure that the observed structures are not an artefact from FRANK. First, we also calculated the radial profiles directly from the synthesised images using the GoFish code^52^. These profiles have less spatial resolution (image synthesis incurs some information loss during the binning and weighting of visibilities) but serve to confirm that substructures are present in the 3-mm image and are not an artefact of FRANK. For this purpose, we produced images with robust = −0.5 to improve the angular resolution. The ring and two gaps are also visible in the image profile. The cavity is only tentatively detected in the profile as a flattening of the intensity in the inner regions.

In addition, we also used the non-parametric GPUVMEM image reconstruction code^63^ to create a maximum-entropy version of the image. The maximum-entropy method implemented in GPUVMEM belongs to the family of regularized maximum likelihood methods, which have gained notable traction in recent years (for example, refs. ^64–66^). The self-calibrated continuum data were super-resolved using GPUVMEM with no entropy term in the objective function^63^; image positivity alone provided sufficient regularization^67^. This approach yielded an image with slightly higher angular resolution and sensitivity compared with an equivalent tclean image. The GPUVMEM image reproduces the same substructures identified in the tclean image while also revealing a small central cavity.

These tests confirm that the inner cavity, ring and gaps recovered by FRANK are real features. The 3-mm robust = −0.5 and the GPUVMEM images, as well as the corresponding radial profiles, are provided in Supplementary Figs. 2 and 3.

### PHANTOM simulations

#### Hydrodynamical simulations

We ran three-dimensional global hydrodynamical simulations using the SPH code PHANTOM^49^, widely used to study gas and dust dynamics in planet-forming environments^68–70^. To model the inner cavity of MP Mus, we ran a grid of 25 simulations varying the semi-major axis and the mass of the companion within ranges compatible with the Gaia PMa. In all the simulations, the mass of the star is *M*_⋆_ = 1.3 *M*_⊙_ and the disk mass is *M*_disk_ = 0.01 *M*_⊙_ (informed by the dust mass calculated from the 3-mm emission and adopting a standard gas-to-dust ratio of 100; Supplementary Information section 1.1). The disk is locally isothermal with a mid-plane temperature profile *T* ∝ *R*^−0.5^, and the initial surface density profile follows *Σ* ∝ *R*^−1^. We adopted a typical disk aspect ratio *H*/*R* to 0.1 (where *H* is the gas scale height) at a reference radius of 100 au (refs. ^71,72^). The resolution is set by the number of particles (*N* = 10^6^). The outer radius is initialized to 50 au as traced by the 1.3-mm continuum observations, and the inner radius is chosen according to the semi-major axis of the inner companion *a*_p_, namely *R*_in_ = 1.5*a*_p_. The central star and the companion were modelled as sink particles, with accretion radii *R*_acc,⋆_ = 0.75*a*_p_ for the central star and *R*_acc,p_ = 0.25*R*_h_ for the secondary, where *R*_h_ is the companion Hill radius. The choice of the primary sink radius is safe because it is always smaller than the semi-major axis of the companion, and the radius of cavity we observe is always three to four times bigger, meaning that it is carved by the tidal interaction rather than being a numerical artefact. We used SPH shock capturing viscosity to model the mechanisms transporting the angular momentum through the disk, prescribing shock capturing viscosity coefficients *α*_AV_ = 0.2 and *β*_AV_ = 2 that resulted in a viscosity parameter of *α*_ss_ = 5 × 10^−3^.

The dust component is set by adopting an initial gas-to-dust ratio of 100 uniformly across the entire disk. We simulated five dust grains sizes ranging from 1 μm to 0.5 cm and with intrinsic density 3 g cm^−3^. The dust is treated with the mixture algorithm, also known as one fluid algorithm (Laibe Price), suitable for coupled dust grains. The mass of the five different grain size populations is calculated according to *n*(*s*) ∝ *s*^−3.5^, where *s* is the grain size.

The mass and semi-major axis of the companion are *M*_p_ ∈ (3, 5, 7, 10, 15) *M*_jup_ and *a*_p_ ∈ (0.5, 1, 1.5, 2, 2.5) au, as discussed in the ‘The Gaia proper motion anomaly of MP Mus’ section. We let the simulations evolve for 1,000 planet orbits, enough time to ensure that the inner cavity reaches a steady state^48^.

#### Radiative transfer

The thermal structure and the 1.3-mm and 3-mm continuum emission images were computed using the Monte Carlo radiative transfer code MCFOST^50,51^. The code takes as input the distribution of gas and dust grains from the hydrodynamical simulation to create a Voronoi mesh where each cell corresponds to an SPH particle. The disk receives passive heating from the central star, modelled as a black body with temperature of 5,000 K, radius *R*_⋆_ = 1.25 *R*_⊙_ and mass *M*_⋆_ = 1.3 *M*_⊙_. We adopted a DIANA standard dust composition^73,74^, that is, 60% silicates, 15% amorphous carbon (optical constants from refs. ^75,76^, respectively) and 25% porosity. The code assumes that submicrometre grains follow the gas, while the spatial distribution of dust grains between micrometres and centimetres is obtained by interpolation. We discretize the radiation field using *N*_γ_ = 10^8^ photons to compute the temperature structure of the disk assuming local thermal equilibrium. Images are then computed via ray tracing again with *N*_γ_ = 10^8^ photons.

## Supplementary information

Supplementary Information Supplementary discussion and Figs. 1–4.

## Data Availability

All the data used in this work are publicly available through the ALMA archive (programmes 2022.1.01758.S and 2017.1.01167.S) and the Gaia archive.

## Code availability

The Python packages used in this study are publicly available via GitHub at ASTROMET (https://github.com/zpenoyre/astromet.py), FRANK (https://github.com/discsim/frank), PHANTOM (https://github.com/danieljprice/phantom), MCFOST (https://github.com/cpinte/mcfost) and GPUVMEM (https://github.com/miguelcarcamov/gpuvmem).
