# Supplementary material for: A young gas giant and hidden substructures in a protoplanetary disk
Source: Nat Astron. 2025 Jul 14;9(8):1176–83. doi: 10.1038/s41550-025-02576-w (PMC12360948; doi:10.1038/s41550-025-02576-w)
Supplement: Supplementary file 1 — Supplementary discussion and Figs. 1–4. [file 41550_2025_2576_MOESM1_ESM.pdf]

nature astronomy

Supplementary information https://doi.org/10.1038/s41550-025-02576-w

A young gas giant and hidden substructures in a protoplanetary disk

In the format provided by the authors and unedited

1 Supplementary material

1.1 Properties and additional analysis of the ALMA data at 3 mm

We measured a 3 mm flux of 22±1 mJy from the image synthesised with `robust=0.5`. The uncertainty is largely dominated by the absolute calibration (5% in Band 3, see the ALMA Technical Handbook). We then calculated a dust disc mass of $0.12^{+0.03}_{-0.02}$ $M_{\rm Jup}$ using standard assumptions [e.g., 1]: optically thin emission, an average dust temperature of 20 K, and a power-law dependence of the dust opacity with frequency following $\kappa_\nu = \kappa_{230\ \rm GHz}(\nu/230\ \rm GHz)^\beta$, where $\kappa_{230\ \rm GHz}$=2.3 cm^2 g^{-1} and β values between 0 and 0.5, as typically inferred in protoplanetary discs [2]. To determine the disc mass and its uncertainty we bootstrapped 1000 estimates by varying the 3 mm flux within its uncertainty, as well as the temperature value following a Normal distribution with a standard deviation of 2.5 K, and β with a uniform distribution between 0 and 0.5. The reported dust mass and uncertainties correspond to the median and the 16% and 84% values. This mass is compatible with the dust mass derived from the 1.3 mm flux in [3].

The radii encompassing 68%, 90%, and 95% of the 3 mm continuum flux are 22±5 au, 35±5 au, and 44±5 au respectively, based on the cumulative curve of the intensity profiles. We used the `robust=0` images for this purpose as they provide a good compromise between angular resolution and sensitivity, but the results with different weightings are compatible within beam size of the observations. The comparison with the $R_{68\,\%}$ and $R_{90\,\%}$ values at 1.3 mm reported in [3] also shows that the disc appears $\sim$30% more compact at 3 mm.

The 3 mm `robust=-0.5` image using `tclean` in `CASA` and the one produced by GPUVMEM and convolved with a similar beam are presented in Supplementary Fig. 1. Supplementary Fig. 2 shows the residuals of the FRANK fit for both wavelengths. These were obtained by first subtracting the FRANK visibilities from the observed ones, and then imaging the result with `robust=0.0`. Finally, the radial profiles calculated from the `robust=-0.5` and GPUVMEM images using GoFish [4] are shown in Supplementary Fig. 3.

1.2 Full set of PHANTOM simulations

Supplementary Fig.4 shows the corresponding radial profiles for the full grid of PHANTOM models. As mentioned in Section 2.3, companions at separations <1 au do not open a large enough cavity, while separations $\geq$2 au carve cavities that are too wide. Increasing the mass of the planet results in deeper cavities. It is also worth noticing that a 3-7 $M_{\rm Jup}$ companion at $\sim$1 au creates a cavity at 3 mm that remains undetected at 1.3 mm, just as observed in the ALMA data. However, this is highly sensitive to the disc and dust properties which we have not explored in detail, and should therefore be considered as a strong indication that planets with such properties can explain the observations, rather than as precise measurements of the planet location and mass.

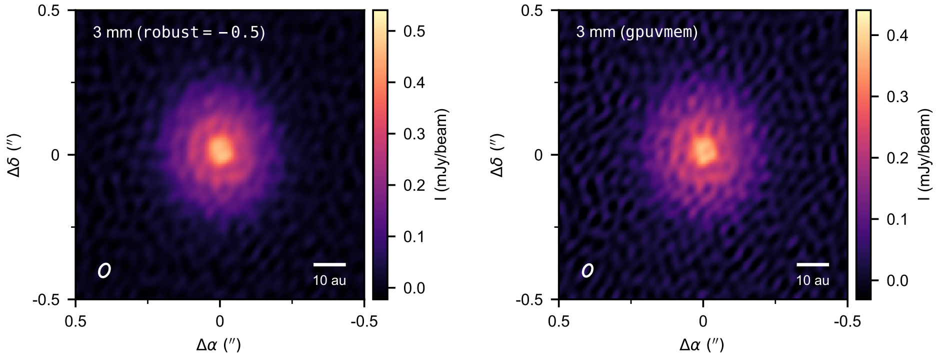

Supplementary Fig. 1 ALMA 3 mm observations of MP Mus synthesised with higher angular resolution. The left panel shows the result of the TCLEAN algorithm with `robust=-0.5`, and the image using the GPUVMEM code after convolution with a similar beam is shown on the right panel. This results in noisier images but improves the angular resolution (0.05"×0.03"). The inner cavity, ring, and gaps are more clearly visible.

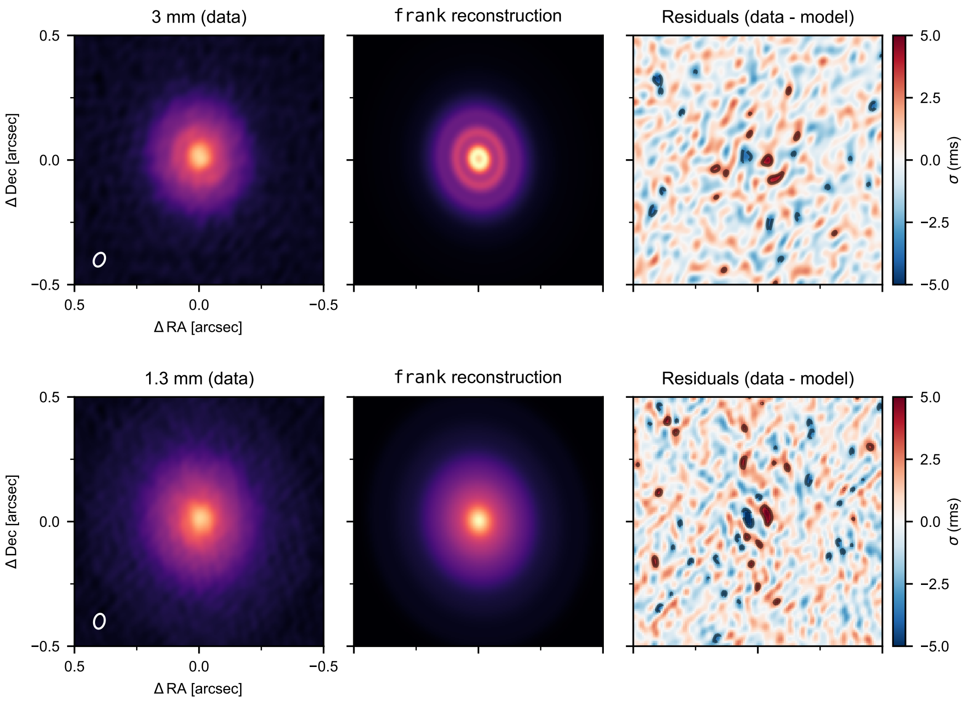

Supplementary Fig. 2 Residuals of the FRANK radial profiles at 3 mm and 1.3 mm. For each case (3 mm top row, 1.3 mm bottom row) the observations are shown on the left, the axisymmetric disc reconstructed from the FRANK profile in the middle, and the residuals appear on the right. Contours are show at ±3 and ±5 times the noise level. Only two localised blobs of ∼5 rms appear in the 1.3 mm data, while no residuals are found above 3 rms in the 3 mm case.

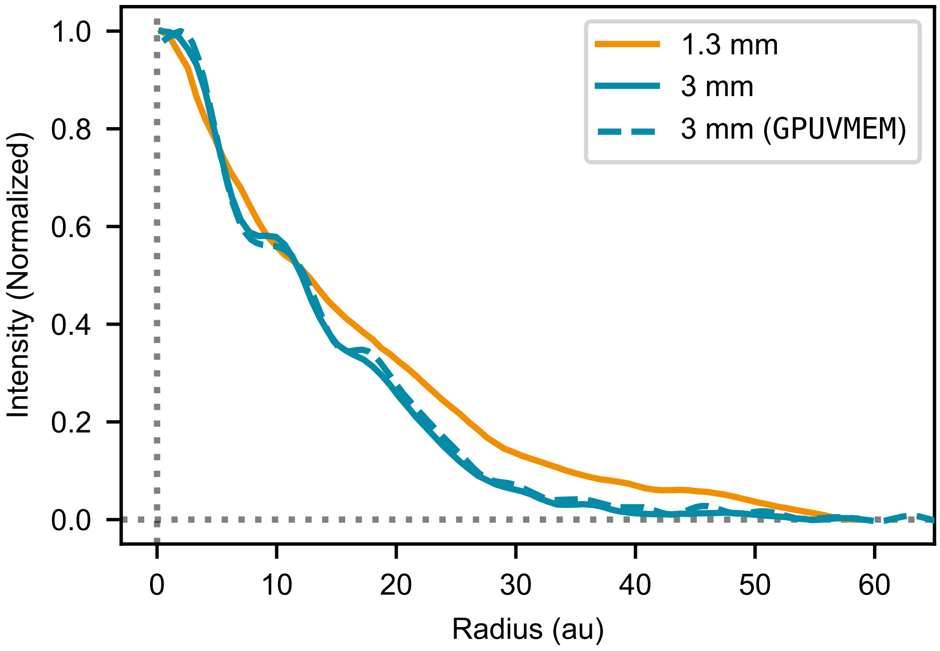

Supplementary Fig. 3 Intensity radial profiles of MP Mus at 1.3 and 3 mm calculated directly from images. The profiles are calculated using the `robust=-0.5` image (solid lines) and the image from GPUVMEM (dashed line) are shown. While the profile of the 1.3 mm appears mostly smooth, the 10.5 au ring and the two gaps at 7.5 and 15 au are clearly visible in the 3 mm one. Although not as clear, the presence of the inner cavity at 3 mm is reflected by the flattening of the radial profile in the inner region.

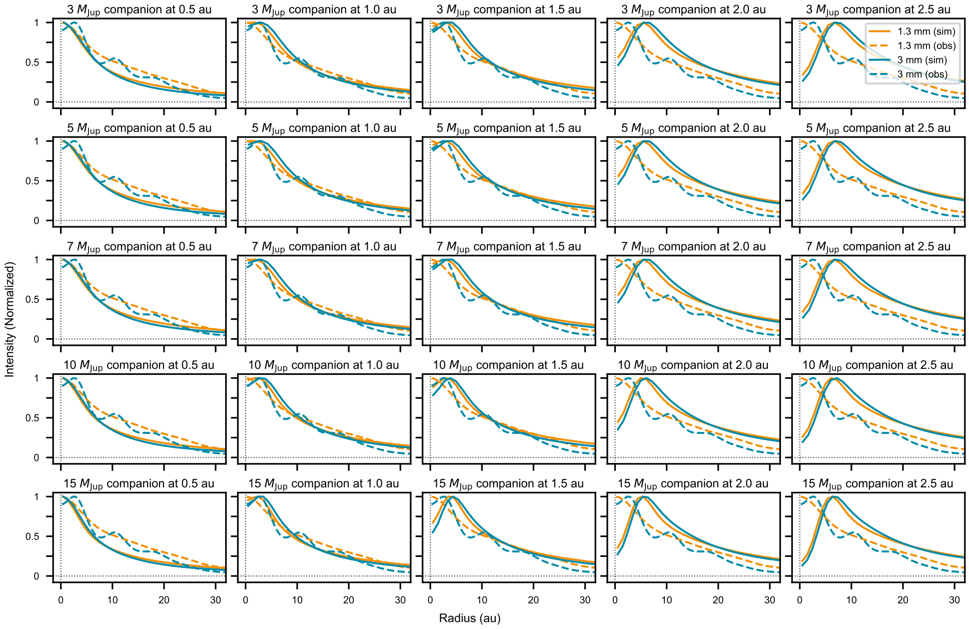

Supplementary Fig. 4 Intensity radial profiles for the full grid of PHANTOM models. Profiles for the simulations (solid lines) and the observations (dashed lines) are shown at 1.3 mm (orange) and 3 mm (blue).

References

[1] Andrews, S. M. & Williams, J. P. Circumstellar Dust Disks in Taurus-Auriga: The Submillimeter Perspective. *Astrophys. J.* **631**, 1134–1160 (2005).

[2] Tazzari, M. *et al.* Multiwavelength continuum sizes of protoplanetary discs: scaling relations and implications for grain growth and radial drift. *Mon. Not. R. Astron. Soc.* **506**, 2804–2823 (2021).

[3] Ribas, Á. *et al.* The ALMA view of MP Mus (PDS 66): A protoplanetary disk with no visible gaps down to 4 au scales. *Astron. Astrophys.* **673**, A77 (2023).

[4] Teague, R. Gofish: Fishing for line observations in protoplanetary disks. *The Journal of Open Source Software* **4**, 1632 (2019). URL https://doi.org/10.21105/joss.01632.
